# Efficacy of a Multicomponent Positive Psychology Self-Help Intervention: Study Protocol of a Randomized Controlled Trial

**DOI:** 10.2196/resprot.4162

**Published:** 2015-08-20

**Authors:** Marijke Schotanus-Dijkstra, Constance HC Drossaert, Marcel E Pieterse, Jan A Walburg, Ernst T Bohlmeijer

**Affiliations:** ^1^ Trimbos Institute Netherlands Institute of Mental Health and Addiction Utrecht Netherlands; ^2^ Centre for eHealth and Well-being Research Department of Psychology, Health and Technology University of Twente Enschede Netherlands

**Keywords:** well-being, flourishing, mental-health promotion, positive psychology, self-help, email support

## Abstract

**Background:**

Positive psychology interventions have been found to enhance well-being and decrease clinical symptomatology. However, it is still unknown how flourishing can also be increased. Although multicomponent interventions seem to be necessary for this purpose, different formats can be used. A cost-effective approach could be a positive psychology-based self-help book with tailored email support to reach large target groups and to prevent dropout.

**Objective:**

This study will evaluate the efficacy of a comprehensive multicomponent self-help intervention with or without email support on well-being and flourishing, and will seek to determine the working mechanisms underlying the intervention.

**Methods:**

In this 3-armed, parallel, randomized controlled trial, 396 participants with low or moderate levels of well-being and without clinical symptomatology will be randomly assigned to (1) a self-help book condition with weekly email support, (2) a self-help book condition without email support but with a weekly information email, or (3) a waiting list control condition. Online measurements will be assessed at baseline, at post-test (3 months after baseline), and at 6 and 12 months after baseline.

**Results:**

The primary outcomes are well-being and flourishing (ie, high levels of well-being). Secondary outcomes are the well-being components included in the intervention: positive emotion, use of strengths, optimism, self-compassion, resilience, and positive relations. Other measures include depressive and anxiety symptoms, personality traits, direct medical and non-medical costs, life-events, and client satisfaction.

**Conclusions:**

This study will add knowledge to the efficacy and cost-effectiveness of a multicomponent positive psychology intervention. We will also explore who can benefit most from this intervention. If the intervention is found to be effective, our results will be especially relevant for public mental health services, governments, and primary care.

**Trial Registration:**

The Netherlands Trial Register NTR4297; http://www.trialregister.nl/trialreg/admin/rctview.asp?TC=4297 (Archived by WebCite at http://webcitation.org/6Uwb5SUUM).

##  Introduction

### Background

Since the introduction of positive psychology in 1998 [1], the proportion of published studies on this topic has grown rapidly each year compared to the entire field of psychological research [2]. Researchers have argued that the study of positive psychology is more capacious than the field of psychopathology because its primary focus is not on dysfunction but on well-being and optimal functioning [3]. Recent evidence indicates that positive mental health and mental illness are related but different continua [4-6]. This so-called two-continua model implies that individuals can experience positive outcomes such as life-satisfaction, meaning, and personal growth when mentally ill, and that the absence of mental illness does not automatically imply high levels of positive mental health [7,8]. As a consequence, researchers have underscored the importance of enhancing positive mental health in addition to preventing and treating mental illness, in clinical populations as well as in the general population [3,9].

A central aim in positive psychology is to increase the amount of flourishing worldwide [1,10]. Flourishing is defined as the presence of high levels of the affective or “feeling good” dimension of well-being (ie, hedonic well-being) in combination with high levels of the psychological functioning or “living well” dimension of well-being (ie, eudaimonic well-being) [4,9,11]. Compared to “languishers” (ie, people at the lowest levels of hedonic and eudaimonic well-being) and individuals with depression, research has shown that flourishers experience the least emotional distress and psychosocial impairment, have hardly any lost work days, and are most likely to survive [4,8]. Keyes and colleagues [12] have found initial support for the hypothesis that an increase in well-being protects against the development of mental illness. More specifically, they found that the risk for having a mental disorder was equally lowest for individuals who were flourishing in 1995 and in 2005, and for individuals who were flourishing in 2005 but not in 1995 [12]. The same study also indicates that it is possible to enhance the amount of flourishing [12]. However, there seems to be significant room for improvement because epidemiological studies using the same operationalization of flourishing have found that there were only 17% flourishers in the United States [4], 20% flourishers in South Africa [13], and 37% flourishers in The Netherlands [14]. Some researchers theorize that even small improvements in the level of well-being in the general population could generate large preventive effects on the amount of psychopathology [9,15]. Yet, little is known about how the amount of flourishing can be increased. The present study will evaluate a theory- and research- based positive psychology intervention (PPI) on efficacy, specific working mechanisms, and cost-effectiveness for enhancing well-being and flourishing in the general population.

### Positive Psychology Interventions

A large number of theories have been used to develop interventions and exercises within the positive psychology movement, as can be found in the Key Competence Happiness Database [16]. Two meta-analyses have shown that PPIs significantly enhance well-being and alleviate depression in the short term, although effect sizes were relatively small [17,18]. These small effect sizes might be a result of the nature and intensity of the included interventions, which were mostly single-component interventions (ie, one or more individual exercises targeting one component of well-being). Examples are “the three good things exercise” or “savoring” targeting positive emotion, and “using one’s strengths in new ways” to increase flow. However, since well-being is a multifaceted construct [10,19] it might be more efficacious to promote well-being and flourishing with a multicomponent intervention that contains a variety of evidence-based individual exercises targeting 2 or more theoretically relevant hedonic and eudaimonic well-being components. For example, the well-being theory of Seligman [10] states that the components of positive emotion, engagement, relationships, meaning, and accomplishment (PERMA) are all necessary for flourishing. Also, multicomponent interventions are higher intensity, target more resources of well-being in an individual, and are often of longer duration than single-component interventions, which may increase their efficacy.

In the last decade, some multicomponent PPIs have been investigated, including different face-to-face interventions. Examples are (1) Positive Psychotherapy, a structured 14-session program developed for individual or group therapy in clinical settings based on Seligman’s well-being theory [10,20,21], (2) Well-being therapy, an individual psychotherapeutic strategy of 8-12 sessions that is based on Ryff’s model of psychological well-being [19] and therefore contains strategies and therapeutic techniques to influence the components self-acceptance, environmental mastery, positive relations with others, personal growth, autonomy, and purpose in life [22], and (3) the Working for Wellness Program, a structured 6-week group-based program for employees with a strong focus on strengths, flow, and social relationships [23]. The efficacy of these multicomponent PPIs on mental illness and positive mental health have been demonstrated in a wide variety of studies—including randomized controlled trials (RCTs)—although most of these studies used small samples [20,22,23]. However, the need for trained therapists in these intensive face-to-face PPIs is a disadvantage that may limit their feasibility, cost-effectiveness and dissemination.

Positive psychology exercises have the advantage of being relatively small and easy-to-implement in daily life, and are therefore well-suited for self-administered interventions. Recently, some Web-based multicomponent PPIs have been developed to increase the reach of PPIs. For example, Schueller and Parks [24] selected 6 exercises from the Positive Psychotherapy intervention (active-constructive responding, gratitude visit, life summary, three good things, savoring, and strengths) and randomly assigned participants to a 6-week program containing all 6 exercises, 4 of the exercises, or 2 of the exercises. The 2- and 4-exercise programs resulted in significantly larger effects on depressive symptomatology than the control group and the 6-exercise program, although participants in the latter program practiced the exercises on significantly more days than participants in the 2- or 4-exercise programs [24]. Another, more comprehensive intervention is Psyfit. This intervention contains 6 modules, each with 4 lessons, about goal setting, positive emotion, positive relations, mindfulness, optimism, and mastery [25]. An RCT showed significant but small effects on well-being and depressive symptomatology, although most participants completed less than 4 lessons of 1 or more modules [25]. While these and other Web-based multicomponent PPIs have shown that they can reach many individuals, their cost-efficacy seem to be limited by low adherence rates [24-27], which could be due to the lack of human contact and coaching [27,28].

### Self-Help Book With Email Support

Another, possibly cost-effective approach could be to intertwine the advantages of the aforementioned formats by using an accessible self-help book in combination with email support to reach and stimulate large groups of individuals at minimal costs. Meta-analyses have shown that the use of self-help books—also called bibliotherapy—with or without support are effective in the treatment of depression [29,30] and the treatment of alcohol problems [31]. Also, research on guided self-help in the form of email support in combination with face-to-face therapy or online therapy has shown promising results [32,33]. Besides, there are some indications that tailored email support is more effective than no support or automated email support [32,34]. More important, promising findings have been found for the use of positive psychology-based self-help books without any support [35-37] or with email support [38]. For example, Parks and Szanto [37] compared a PPI group of students who received the book “The How of Happiness” [39] to a group of students who received a cognitive behavioral self-help book to cope with depression and a control group. Results showed that both intervention groups were equally effective in reducing depressive symptoms at post-test compared to the control group, and that significant increases in life-satisfaction were found only for the PPI compared to the control condition at the 6-month follow-up. Another study used a multicomponent self-help book based on acceptance and commitment therapy in a sample with mild to moderate depressive symptomatology [38]. One intervention group received the book with extensive and tailored email support on progress and process, while another group received the book with minimal email support on progress. Results revealed significant increases in well-being and mindfulness and significant decreases in depressive, anxiety, and fatigue symptomatology for both intervention groups at 3-months follow-up compared to a waiting list control group [38]. In this study, we will use a version of the extensive email support similar to that used in the latter study [38]. That is, participants will be guided through the self-help book by tailored email feedback from trained counselors, but we will compare it to receiving an information email instead of minimal email support.

### This Study

This study will examine a comprehensive multicomponent positive psychology-based self-help book with email support. The self-help book is entitled, “This is your life” (TL) [40] and targets 6 key components in positive psychology: positive emotion, use of strengths, optimism, self-compassion, resilience, and positive relations. We will compare a condition wherein participants receive the book with weekly asynchronous and tailored email support (TL-E) to a condition wherein participants receive the book with a weekly email with information (TL-I). Both groups will be compared to a waiting list control condition (WL). Our research aim is to evaluate the efficacy of the multicomponent self-help book with or without email support in terms of well-being and flourishing in an RCT (NTR4297) and to evaluate which of the included well-being components in the self-help book contribute to its efficacy. We expect that TL-E will be equivalent or superior to TL-I and superior to WL at post-test and 6 months follow-up, and that the effects will be sustained at 12 months follow-up.

This study will add to current knowledge in 3 ways. First, it will be conducted in a sample with low or moderate levels of well-being without moderate or severe clinical symptomatology, which is a potential “happiness-seekers” group [41] that has not previously been studied in PPIs [17,18,27]. Second, this study will gain insight into the working mechanisms underlying the intervention by evaluating which of the included well-being components in the self-help book (ie, positive emotion, use of strengths, optimism, self-compassion, resilience, and positive relations) contribute to its efficacy and by examining individual differences in demographic characteristics, number of life events, personality traits, and adherence rates. Third, this study will examine if the innovative format used in this study (ie, self-help book with relatively inexpensive email support) is a cost-effective approach for enhancing well-being and flourishing compared to a waiting list control group, since little is known about the cost-effectiveness of PPIs [26].

## Methods

### Study Design

This study is a parallel RCT with 3 groups: a group receiving the self-help book with weekly asynchronous and tailored email support (TL-E); a group receiving the self-help book without email support but with a weekly information email (TL-I); and a waiting list control group (WL). Outcomes will be assessed at baseline (T0), post-test (around 3 months after baseline; T1), and at 6 (T2) and 12 months (T3) after baseline. All measurements will be self-reported and gathered via email with a link to a personal questionnaire built with Qualtrics software [42]. The design and procedures of this study were approved by the Ethics Committee of the University of Twente in The Netherlands (no. 13212).

### Participants

Participants will be recruited from the general population in The Netherlands using newspaper advertisements. The recruitment messages will be positively framed: (1) “Get the best out of yourself and improve your resilience and well-being with a free self-help course”, and (2) “Become happy and stay happy? Improve your resilience and well-being with a free self-help course”.

**Figure 1:**
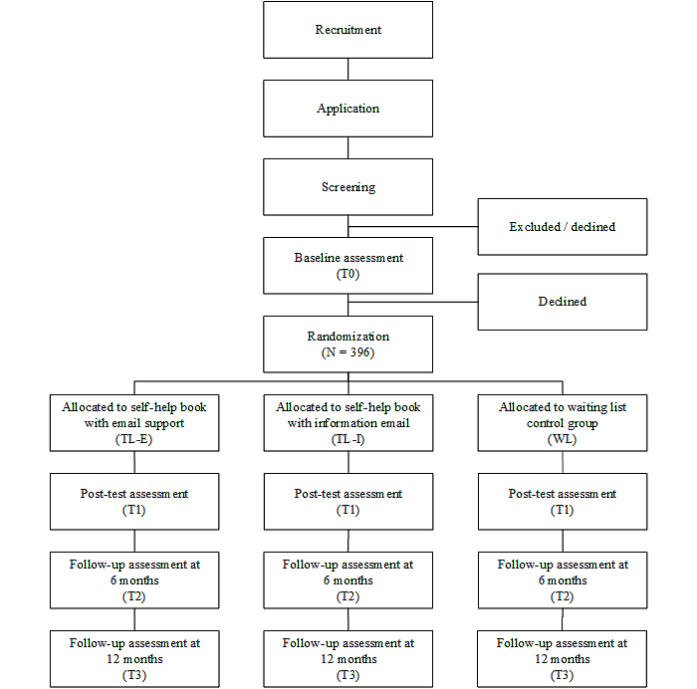
Design of the study and intended flow-chart of participants.

Interested participants will be directed to a website with extensive information about the study and an application form. After applying, participants will be sent a personal link to a screening questionnaire on their specified email address. Participants who open the personal link will be asked to give online informed consent. If no informed consent is given, participants will be diverted to the information website and will not be able to participate in the study. If informed consent is given, participants will fill out the screening questionnaire before the start of the study. Figure 1 shows the intended flow-chart of participants.

Adults aged 18 or above with low or moderate levels of well-being without moderate or severe clinical symptomatology are eligible for this study because previous studies have shown that these individuals might search for happiness [41] and could boost their mental health by elevating the level of well-being [7,8]. Other inclusion criteria are a sufficient internet connection, a valid email address, and a willingness to invest an average of 4 hours per week for 8-12 weeks. In the screening questionnaire, the Mental Health Continuum Short Form (MHC-SF) will be used to assess the level of well-being and the Hospital Anxiety and Depression Scale (HADS) will be used to assess the level of clinical symptomatology. Participants will be excluded from the study if they score 4 or 5 (range 0-5) on at least 1 of the 3 emotional well-being items in combination with a score of 4 or 5 (range 0-5) on at least 6 of the 11 social and psychological well-being items of the MHC-SF, which is operationalized as flourishing (ie, high levels of well-being) [13,43]. Participants will also be excluded if they score 10 or higher (range 0-21) on the depression scale and/or on the anxiety scale of the HADS, which indicates individuals with moderate or severe clinical symptomatology [44]. Participants who will be excluded from the study will be notified by email because, (1) they are already flourishing, or (2) they probably have at least moderate depression or anxiety symptoms. The latter group will receive their specific scores and their meaning on the HADS and will be advised to talk to their general practitioner when symptoms persist or increase. After screening, the remaining participants receive a personal email link to the baseline questionnaire.

### Randomization

Randomization will be conducted after all eligible participants complete the baseline questionnaire, with 396 participants being randomly assigned to 1 of the 3 groups (allocation ratio, 1:1:1). Randomization will be stratified by gender and educational level (ie, low, medium, high) using a computerized random number generator created with Excel. This is an automated process with no interference from the investigators.

### Interventions

This study will use the self-help book, “This is your life” [40] in both experimental conditions. This book is mainly based on the overarching and comprehensive well-being theory of Seligman [10] and Ryff’s theory of psychological well-being [19]. The book consists of 8 modules on 6 key components in positive psychology: positive emotion; discovering and using strengths; optimism and hope; self-compassion; resilience and post-traumatic growth; and positive relations (Table 1). Each module contains psycho-education derived from specific theories and empirical evidence in positive psychology and related research areas. Each module also contains a number of positive psychology exercises, ranging from 3-10 per module. Most of these exercises are evidence-based, such as the "three good things exercise" [17,18] and "imagine your best possible self" [45-47]. All modules are, in theory, effective independent strategies for enhancing well-being. Participants in the experimental groups will receive the self-help book by regular mail, accompanied by a time schedule for reading the book and practicing the recommended exercises (see Table 1). Participants will be instructed to read one module per week—except for module 2, which can be spread out over two weeks—in sequential order, and to practice the recommended exercises of that module. If the participant has more time, he or she can re-read the module and practice other exercises of that module. Participants will be encouraged to invest the most time in the exercises that feel most beneficial to them. Participants will have 8-12 weeks to complete the 8 modules of the book to take holidays and other circumstances into account. When participants in the TL-E group need more than 9 weeks to complete the book, in consultation with the counselor, they will not receive email support in the weeks they are not in the position to work on a module. Participants in the TL-I group will receive weekly information emails during the first 9 weeks and will be asked to read the emails in chronological order in the weeks they actually work on a module. On average, participants in the experimental groups will be expected to invest 4 hours per week during the intervention period.

**Table 1:** Content of “This is your life” and recommended exercises for participants in the experimental conditions.

Module	Recommended exercises	Theoretical background
1. Positive emotions	Diary of pleasant emotions: What happened, who was there, what did you feel, what did you thought?	Fredrickson, 1998 [48]; Fredrickson, 2001 [49]; Fredrickson, 2009 [50]
	Three good things: Think about three things that went well today and savor those moments.	
2. Discovering strengths	Overview of your strengths: Which of the 47 strengths do you have and which of these give you energy and pleasure?	Linley et al., 2010 [51]; Linley and Harrington, 2006 [52]
	Identify your strengths I: Answer the 10 questions (ie, who inspires you?) that will help you to discover your strengths.	
	Identify your strengths II: Which strengths do you recognize in answering the 10 questions?	
	Vision of others: Ask 3-5 people about your top 5 strengths with examples from daily life.	
	Top 5 strengths: Based on all previous exercises, choose your top 5 strengths that also give you energy and pleasure.	
		
3. Use of strengths, flow	Change “must” into “want”: Make a list of things you don’t like but must do. What are underlying intrinsic motivations?	Csikszentmihalyi, 2001 [53]
	Flow: Have you experienced flow and why?	
	Flow at the moment: How much flow did you experience the preceding week? When, how?	
	Challenge yourself: How can you create more flow in your life? Use your strengths in a new way.	
		
4. Optimism, hope	ABC-Diary: What do you think and do when something negative happens? How can you challenge favorite pessimistic thoughts?	Carver et al., 2010 [54]; Scheier and Carver, 1992 [55]; Seligman, 1990 [56]
	Imagine your best possible self: Visualize yourself in the personal, relational, and professional domain.	
		
5. Self-compassion	Wish yourself something good: Be mindful and identify your greatest need at this moment. Use your inner voice to repeat your compassionate wish.	Gilbert, 2009 [57]; Neff, 2003 [58]; Neff and Germer, 2012 [59]
	Develop a compassionate inner voice: Write 5 minutes about situations in the preceding week wherein you showed self-compassion.	
		
6. Resilience	Coping style: Take the test to identify your prominent coping style(s).	Joseph, 2011 [60]; Joseph and Linley, 2006 [61]; Tedeschi and Calhoun, 2004 [62]
	Expressive writing: Write 15 minutes on at least 4 days about emotions, thoughts, and feelings around a negative or positive event.	
	Needs: What are your specific needs at this moment? Who should know your needs?	
		
7. Positive relations (I)	Active-constructive responding: Respond positively to good news shared by others. Use active communication skills, how does the other react?	Gable et al., 2004 [63]; Reis and Gable, 2003 [64]; Reis et al., 2010 [65]; Rosenberg, 2009 [66]
	Listen compassionately: Try to use elements of compassionate listening, such as “What feelings and needs does the other express?”	
	Expressing gratitude: Write a gratitude letter and/or read it aloud to the person you are thankful to.	
		
8. Positive relations (II)	Relaxation/meditation: Relax by doing a “body scan”, physical exercise, or “stand like a tree”.	Bloom, 2011 [67]; Otake et al., 2006 [68]
	Reflect on your needs: What are your intrinsic goals, needs and motives? Do you live those needs and why (not)?	
	Acts of kindness: Rejoice somebody by performing an unexpected act of kindness or by doing volunteer work.	

#### Email Support

The TL-E group will receive weekly email support from a personal counselor. These counselors will be 5 senior positive psychology students of the University of Twente who completed a course on email counseling in which they are trained to guide a fellow student on using the self-help book “This is your life”. Each of these will guide 25 participants and the remaining participants will be guided by the first author (MS). Participants will be randomly assigned to 1 of the 6 counselors in the same manner as the randomization procedure outlined above (eg, stratified by gender and educational level). Additional training will be given in a 1-day workshop and via weekly supervision by authors MS and EB and a clinical psychologist. In these meetings, random emails of the counselors will be discussed to increase treatment integrity, and counselors can ask specific questions to the supervisors. The personal counselors will introduce themselves in an initial email sent the day participants receive the self-help book. The participants will be asked to introduce themselves before the email sessions start. Participants will be instructed to send a weekly email on Sunday or Monday wherein they write about their experiences with the scheduled module. Every Wednesday, the personal counselors will answer the emails.

The main goal of the email support is to encourage the participants to read the scheduled modules and practice the recommended exercises. The email support will be more process-oriented than content focused, and will be flexible within the boundaries of the structured time schedule. The personal counselors will be instructed to provide tailored feedback on progress and process using positive reinforcement, paraphrasing, and motivational interviewing techniques (eg, “I read that you have invested quite some time in the recommended exercises of this week.”; “Could it be helpful for you to practice this exercise more regularly?”; “What have you discovered when you performed the exercises?”). Specifically, counselors will use positive reinforcement for signs of awareness, insights, improvement and change (eg, “This seems a valuable experience for you, how exactly did you achieve that?; “What will be a next sign of progress?”; “That seems like a helpful thought to me.”). When there are reasons to assume that a participant has serious complaints, the participant will be referred to a general practitioner or a health care specialist. In an attempt to reduce intervention dropout, reminders will be sent to participants who do not send an email to their counselor. The first 2 reminders will be sent by the personal counselor, followed by up to 3 additional reminders sent by the researcher (MS). If a participant has completed more than 3 modules of the book, he or she will only receive reminders from their personal counselor. A final email with overall feedback on the process of the participant will be sent to all participants who complete at least 3 modules. This final email will be sent when the participant has completed all modules (between 8 and 12 weeks), when the maximum of 3 reminders has been reached by the personal counselor, or when participants are in week 12 of the study without completing all modules.

#### Email With Information

The TL-I group will receive a weekly email from the investigator that contains additional information about the self-help book or the study. A frequently-asked-questions (FAQs) format will be used for this email (eg, “Q: How can I find the time to practice the recommended exercises?” “A: Select a good moment in your daytime routine, so that practicing the exercises can become a habit. For example, during lunch time, directly after work or half an hour before bed time.”). The information emails will be a work-in-progress during the study because FAQs by participants in the TL-E group will also be integrated into the information emails. Participants will be instructed to read the weekly emails and not to respond to the investigator.

#### Control group

Participants in the control group will be on a waiting list for the first 6 months of the study and will receive the self-help book after they complete the 6-month follow-up assessment. For the sake of comparison at the long-term follow-up, the control group will also receive the information emails in the same way provided in the TL-I group.

### Measures

For a brief overview of all outcome measures and assessment times, see Table 2. Because one of our aims is to examine the working mechanisms underlying the intervention, which requires assessing all included positive psychology components, measures are selected on brevity and availability in the Dutch language.

**Table 2:** Intended questionnaires and assessment times.

			T0	T1	T2	T3
Questionnaire	Measurement	Screening	Pre-test	Post-test	Follow-up^a^	Follow-up^b^
MHC-SF	Well-being/ flourishing	X	X	X	X	X
FS	Social-psychological well-being		X	X	X	X
HADS	Symptoms of depression and anxiety	X			X	X
m-DES	Positive and negative emotions		X	X	X	X^d^
SUS	Use of strengths		X	X	X	X^d^
LOT-R	Optimism		X	X	X	X^d^
SCS-SF	Self-compassion		X	X	X	X^d^
BRS	Resilience		X	X	X	X^d^
SPR	Positive relations		X	X	X	X^d^
EPQ-RSS	Extraversion and neuroticism		X			
NEO-FFI	Conscientiousness		X			
MCQ	Direct medical consumption costs		X	X^c^	X^c^	X
PCQ	Non-medical productivity costs		X			X
Brugha Life-events	Positive and negative life-events		X	X	X	X
CSQ-8	Client satisfaction			X^d^		
Process evaluation	Invested time, adherence, positive changes			X^d^		X^e^
	Level of (continued) use					X
Demographics	Gender, age, education, marital status, living situation, ethnicity, daily activities	X				

^a^ 6 months after T0.

^b^ 12 months after T0.

^c^ Only medical health care costs are assessed, not medication costs.

^d^ Only assessed in the experimental groups, not the control group.

^e^ Only assessed in the control group, not the experimental groups.

#### Primary Outcome

Well-being is the primary outcome of this study, measured with the 14-item MHC-SF and the 8-item Flourishing Scale (FS). The items of the MHC-SF can be divided into 3 subscales: emotional well-being (3 items), social well-being (5 items), and psychological well-being (6 items). All items can be answered on a 6-point answer scale from 0 (never) to 5 (almost always). A mean score will be computed separately for the total scale and the 3 subscales. The MHC-SF has excellent psychometric properties [5,13]. The FS measures social-psychological well-being with 8 positively formulated items. Participants rate the items on a 7-point scale from 1 (strongly disagree) to 7 (strongly agree). Total summed scores can range from 8-56. The FS has demonstrated good internal consistency and construct validity [69].

#### Secondary Outcomes

The MHC-SF is also used to measure the amount of flourishing [13,43]. Flourishers score 4 or 5 on 1 or more items on the emotional well-being subscale in combination with a score of 4 or 5 on 6 of the 11 remaining items. Languishers are those with scores of 0 or 1 on these subscales, and individuals who are neither flourishing nor languishing are labelled as moderately mentally healthy.

Depressive and anxiety symptoms will be measured with the HADS [44]. Each subscale consists of 7 items, 14 items in total. Answer categories differ per item, but all items have 4 answer categories with scores ranging from 0-3. Item scores are summed into a total scale score for anxiety (range 0-21) and depression (range 0-21). The HADS is a widely validated instrument and can be used as a screening instrument in different populations [70,71]. A cut-off score of 10 and above on each subscale is generally used to identify individuals with at least mild depressive or anxiety symptomatology [44,70].

Positive and negative emotional states will be assessed with the modified Differential Emotions Scale (m-DES), which measures 8 groups of positive emotions and 8 groups of negative emotions on a 7-point scale (1 = not at all, 7 = very intense). Total mean scores will be computed. Although the DES is validated and widely used, the m-DES has not yet been validated [72].

The Strength Use Scale (SUS) is a 14-item scale to assess participants’ use of their strengths in a variety of settings [73]. Scores range from 1 (strongly disagree) to 7 (strongly agree) with a total summed score of 14 to 98. The SUS possesses high internal consistency and long-term stability [74].

Optimism will be assessed with the 10-item Life Orientation Test-Revised (LOT-R) [75,76]. Items are rated on a 5-point scale ranging from 0 (strongly disagree) to 4 (strongly agree) and indicate participants’ evaluation of positive expectations about the future. There are 4 filler items which are excluded from analysis. Scores on the 6 items are summed into a total optimism score ranging from 0-24. The LOT-R has shown predictive and discriminant validity [75].

Self-compassion will be assessed with the Self-Compassion Scale-Short Form (SCS-SF), which consists of 12 items. Participants rate the items on a 7-point scale ranging from 1 (rarely or never) to 7 (almost always) with a total summed score ranging from 12-84. The SCS-SF has proven to be a valid equivalent of the long-form version [77] developed by Neff [78].

The 6-item Brief Resilience Scale (BRS) [79] will be used to evaluate the level of resilience, which is the ability to bounce back and cope with stress or negative life-events. Items are rated on a 5-point scale from 1 (strongly disagree) to 5 (strongly agree) with total mean scores ranging from 1 to 5. The psychometric properties are good [79].

Although positive relations will be assessed with 1 item of Ryff’s scales of psychological well-being [19] in the MHC-SF, we will also measure the original Subscale of Positive Relations (SPR) as proposed by Ryff [19]. This subscale contains 9 items and a 6-point scale ranging from 1 (strongly disagree) to 6 (strongly agree) [80] and a total summed score that range of 9-54.

#### Tertiary Outcomes

The personality traits extraversion and neuroticism will be assessed with the Eysenck Personality Questionnaire-Revised Short Scale (EPQ-RSS) [81-83] and conscientiousness with the NEO Five Factor Inventory (NEO-FFI) [84]. These personality surveys are widely used and validated. Direct medical and non-medical costs will be measured with the Dutch Medical Consumption Questionnaire (MCQ) [85] and 5 items from the Productivity Cost Questionnaire (PCQ) [86]. Other variables that will be assessed are positive and negative life-events with a scale based on the Brugha Life-events section [87], and the socio-demographics of gender, age, educational, marital status, living situation, ethnicity, and daily activities.

#### Process Outcomes

The Client Satisfaction Questionnaire-short form (CSQ-8) [88,89] will be used to determine overall satisfaction. Additional questions will be asked about time spent with the intervention and self-help book modules, adherence to each module, and significant positive changes since they started with the intervention. The latter question is based on the Client Change Interview Protocol [90]. All email correspondence between participants and personal counselors will be retrieved and saved for content analysis.

### Sample Size

Well-being is the primary outcome used for the power calculation. We expect to detect a significant effect on well-being between the experimental groups TL-E and TL-I compared to the WL group. Based on recent meta-analyses [17,18] and a previous comparable study [38], we expect to find a standardized effect size on well-being of 0.40 or larger. The targeted sample size is 99 participants per group (297 in total) to provide a statistical power of (1 - *β*) = 80% and a 5% significance level (two-tailed). To allow a study dropout rate of 25%, we need 132 participants in each condition and 396 participants in total for the trial.

### Statistical Analyses

The Consolidated Standards of Reporting Trials (CONSORT) statement [91] will be used to report the results. Independent *t* tests and Chi-square statistics (X^2^) will be used to examine baseline differences between the study groups. Non-significant socio-demographic differences at baseline will indicate successful randomization. Internal consistency of the constructs will be determined using Cronbach’s alpha. To examine the efficacy of the intervention, intention-to-treat analyses will be conducted by including all participants in the analyses who have been randomized. The Expectation Maximization (EM) method will be used to impute all missing data on the continuous measures of T1, T2 and T3. This method imputes the data by maximum likelihood estimation using the observed data in an iterative process [92]. The intention-to-treat analysis will be compared with a completers-only analysis.

To examine significant differences between the 3 groups, we will perform repeated measures ANOVA in a 3 (group) x 3 (time) design with well-being (MHC-SF and FS) as the primary dependent variable and positive/negative emotions (mDES), the use of strengths (SUS), optimism (LOT-R), self-compassion (SCS-SF), resilience (BRS), and positive relations with others (SPR) as the secondary dependent variables. Significant time x group interactions will be tested with Tukey’ post hoc tests. Within-group effects (Cohen’s *d*) will be calculated by subtracting the mean post-test or follow-up score from the mean baseline score and dividing the difference by the pooled standard deviation. Between-group effects (Cohen’s *d*) will be calculated by subtracting the mean post-test or follow-up score of the experimental group from the mean post-test or follow-up score of the control group, and dividing the difference by the pooled standard deviation [93]. These effect sizes are used to gain insight into the relevance of the significant effects. To examine changes in the amount of flourishers, languishers, and moderately mentally healthy (MHC-SF), descriptive statistics will be used. Changes in categories will be tested with X^2^-statistics.

Mediator and moderator analyses will be performed as described by Hayes [94,95]. Mediator analyses will be conducted to investigate which of the specific components of the intervention—positive emotion, use of strengths, optimism, self-compassion, resilience, and positive relations—contribute to its efficacy and mediate overall well-being. Moderator analyses will be conducted to examine which specific subgroups will improve most (or least) on well-being and flourishing from participation in one of the experimental groups. The independent variables of gender, educational level, life-events, personality traits, level of adherence to the intervention, and participation satisfaction will be entered as moderators in regression analyses.

A cost-effectiveness analysis will be conducted from a societal perspective. For each condition, the mean annual health care costs (MCQ), costs due to productivity losses (PCQ), and intervention costs will be calculated on an annual per capita basis for the reference year 2014. All costs will be expressed in Euro (€). The incremental cost-effectiveness ratio (ICER) will be calculated using a bootstrap approach. Then, a cost-effectiveness acceptability curve will demonstrate the probability of the cost-effectiveness of TL-E and TL-I compared to WL, for a range of willingness-to-pay (WTP) ceilings. All tests will be two-sided and will use alphas of 0.05. All data-analyses will be performed with SPSS version 21.0 or higher.

## Results

The data collection will be completed in June 2015.

##  Discussion

This study will investigate the short- and long-term efficacy of a multicomponent PPI (ie, self-help book with tailored email support) on well-being and flourishing. Mediator and moderator analyses will be conducted to explore which theoretically relevant hedonic well-being and eudaimonic well-being components included in the self-help book appear to contribute to its efficacy, and who will benefit most from the intervention. The study will be conducted in The Netherlands in a sample with low or moderate levels of well-being without moderate or severe clinical symptomatology. The results from this study will contribute to the knowledge of evidence-based PPIs, in particular multicomponent PPIs, and will be valuable for public mental health services and governments who are increasingly interested in enhancing well-being in the general population [96,97].

There could be several limitations that need to be considered in advance. First, our results might not be generalizable to the general Dutch population, since we will use a self-selected sample and apply exclusion criteria on the level of well-being and clinical symptomatology. It is not unlikely that our open recruitment with advertisement in national newspapers will attract mostly highly-educated women. However, we will use a randomization procedure stratified by gender and education, which will create equal distribution of these characteristics to each group and therefore not harm the internal validity of our study. Second, non-adherence to the intervention and drop out from assessments (incomplete data) could occur. Both types of attrition are closely related and may bias our results [98]. Reasons for non-adherence and dropout could be related to individual characteristics (ie, low-educated individuals might find the intervention and/or assessments too difficult), characteristics of the intervention (ie, individuals in the experimental conditions receive the self-help book after baseline and are unaware of the content of the book and type of exercises when they apply for the study), and characteristics of the study (ie, participants will be given instructions and a time schedule) [98]. To gain insight on adherence in our study, participants will be asked about time spent on each module, and the personal counselors will gather reasons for non-adherence mentioned in the email correspondence. Although we expect to find higher adherence rates for individuals in the TL-E condition than has previously been found for Web-based multicomponent PPIs [24,25,27], it could be lower than in face-to-face multicomponent PPIs [20,22,23]. However, our study can only draw conclusions about the comparison of the self-help book with email support to the self-help book without email support. Differences in adherence and efficacy between face-to-face support, email support, and no support warrant attention in future research. Furthermore, to prevent low power due to drop out, we have taken into account an attrition rate of 25% in our power analysis. We will also conduct dropout analysis to identify any selectiveness or characteristics of attrition that may be relevant for real-life implementation of the intervention. Third, the use of senior positive psychology students for the email support can be a risk for the professionalism of our intervention because these students are unexperienced psychologists. We will try to reduce this risk by providing extensive supervision from experienced psychologists and by only selecting students who are thoroughly trained in email support and who have practiced the self-help book. On the other hand, the guidance is primarily process-oriented and advanced clinical counseling skills are therefore not considered necessary. The benefit of using students as counselors is that the intervention costs will be very low. If proven effective, it could be even more effective when implemented in a mental health service with experienced and professional counselors, although this could be disadvantageous for its cost-effectivity. Nevertheless, this needs to be tested in future research.

Our study will be the first to investigate the efficacy and working mechanisms of a theory- and research- based multicomponent PPI in the form of a self-help book with email support to enhance well-being and flourishing in the general population. Our study will add to the existing knowledge in that we (1) focus on well-being and flourishing instead of mental illness, (2) use a multicomponent intervention instead of a single-component intervention, (3), integrate some advantages of face-to-face multicomponent PPIs with web-based multicomponent PPIs by using a self-help book with asynchronous and tailored email support, (4) examine the intervention in a population with low or moderate levels of well-being without moderate or severe clinical symptomatology instead of a population with at least moderate clinical symptomatology without any criteria on the well-being dimension of the two-continua model of mental health, and (5) measure and analyze each specific well-being component that is included in the self-help book: positive emotion, use of strengths, optimism, self-compassion, resilience, and positive relations. Taken together, our results will contribute to a new field of multicomponent PPIs for enhancing well-being and flourishing in the general population and highlight a new target population for mental health promotion. If proven effective, our results can be specifically of interest for public mental health and primary care because these health care fields are expanding, while specialist mental health care is diminishing, at least in The Netherlands.

## Abbreviations

ANOVA: analysis of variance
BRS: Brief Resilience Scale
CONSORT: Consolidated Standards for Reporting Trials
CSQ-8: Client Satisfaction Questionnaire-short form
EM: expectation maximization
EPQ-RSS: Eysenck Personality Questionnaire–Revised Short Scale
FS: Flourishing Scale
HADS: Hospital Anxiety and Depression Scale
ICER: incremental cost-effectiveness ratio
LOT-R: Life Orientation Test-Revised
MCQ: Medical Consumption Questionnaire
m-DES: modified Differential Emotions Scale
MHC-SF: Mental Health Continuum-Short Form
NEO-FFI: NEO Five Factor Inventory
PCQ: Productivity Cost Questionnaire
PERMA: positive emotion, engagement, relationships, meaning and accomplishment
PPI: positive psychology intervention
RCT: randomized controlled trial
SCS-SF: Self-Compassion Scale-Short Form
SPR: Subscale of Positive Relations
SPSS: Statistical Package for the Social Sciences
SUS: Strengths Use Scale
TL: “This is your life”
TL-E: “This is your life” with email support
TL-I: “This is your life” with an information email
WL: waiting list control condition
WTP: willingness-to-pay
